# First-person visual perspective enhances audiovisual synchrony effects in autonomous sensory meridian response

**DOI:** 10.3389/fpsyg.2026.1740614

**Published:** 2026-03-27

**Authors:** Nodoka Sakakihara, Ryo Kitada

**Affiliations:** Graduate School of Intercultural Studies, Kobe University, Kobe, Japan

**Keywords:** ASMR, audition, emotion, multisensory interaction, social touch, vision

## Abstract

Autonomous sensory meridian response (ASMR) is a tingling sensation at the scalp and moving down the neck, which is often accompanied by positive feelings. ASMR is typically induced by auditory stimulation produced by someone’s actions, but studies suggest that such auditory-evoked ASMR can be modulated by visual input. What factors contribute to audiovisual interactions that generate ASMR? Although studies have shown that audiovisual congruency plays a role, the contributions of other visual factors, such as perspective and proximity, remain poorly understood. Here, we conducted two preregistered psychophysical experiments to examine the effects of visual perspective and audiovisual congruency on ASMR. Participants rated the intensity, valence, and arousal of experienced ASMR after watching video clips in which audiovisual temporal synchrony was manipulated. In Experiment 1, participants observed a dummy head being stimulated with objects by another person (third-person perspective), and audiovisual congruency showed no significant effects across perceptual dimensions. In Experiment 2, where both audiovisual congruency and visual perspective were manipulated, the congruency effect of the intensity emerged only when participants observed the actions from a first-person perspective. These results highlight the critical role of visual perspective in visuo-auditory interactions underlying ASMR evoked by others’ action sounds.

## Introduction

In daily life, we watch and listen to various objects that produce sounds, which in turn evoke diverse emotions and bodily sensations. Among these experiences, one particular kind can elicit tingling sensations accompanied by positive feelings known as autonomous sensory meridian response (ASMR). ASMR is a sensory–emotional phenomenon characterized by tingling sensations, that is typically beginning at the scalp and moving down the neck and spine (Ahuja, 2013; Barratt and Davis, 2015; Poerio et al., 2023). ASMR is triggered by specific audiovisual stimuli (“triggers”) such as whispering, tapping, personal attention, and slow movements (Barratt and Davis, 2015; Barratt et al., 2017). ASMR shares some similarities with synesthesia and frisson (music-induced chills, Kovacevich and Huron, 2018) but is distinct in its trigger profile and response characteristics (Mahady et al., 2023). Individual sensitivity to ASMR has been linked to personality traits such as higher openness and neuroticism, and lower extraversion and conscientiousness (Fredborg et al., 2017; Janik McErlean and Banissy, 2017). Interest in academic research on ASMR has grown because ASMR is reliably identified through self-reported experiences (Barratt and Davis, 2015) and its positive affective component can help alleviate distress (Barratt and Davis, 2015; Fredborg et al., 2018; Poerio et al., 2018; Smejka and Wiggs, 2022; Mahady et al., 2023). Despite the growing number of studies, however, the nature and underlying mechanisms of ASMR are not fully understood.

One line of previous studies on ASMR has examined the acoustic characteristics of sounds that trigger ASMR (Barratt et al., 2017; Koumura et al., 2021; Shimokura, 2022; Terashima et al., 2024; Hozaki et al., 2025; Jones et al., 2025). For instance, ASMR intensity increases when participants listen to binaural sound objects moving around the head (Koumura et al., 2021) or to sounds presented close to the ears (Honda et al., 2020; Koumura et al., 2021). Lower-pitched, darker timbres (i.e., deeper, softer voices or sounds) are more effective ASMR triggers (Koumura et al., 2021). Human-generated sounds (e.g., whispering, tapping, brushing, mouth sounds) elicit stronger ASMR responses than nature-generated noises such as rain or wind (Shimokura, 2022). These studies indicate that ASMR sounds tend to be soft, close-miked, and socially evocative, often mimicking tactile interactions and emphasizing lower frequencies and high spatial fidelity. It is proposed that ASMR may replicate comforting effects of social bonding behaviors, such as grooming (Barratt and Davis, 2015; Lochte et al., 2018; Poerio et al., 2018; Hozaki et al., 2025).

In everyday life, however, we rarely rely on audition alone to perceive others’ actions. Several studies have examined the effects of visual stimulation on ASMR (Fredborg et al., 2021; Maeno and Kajimura, 2022; Kim et al., 2024; Hozaki et al., 2025; Jones et al., 2025). For instance, although ASMR occurrence and intensity under the perception of visual stimuli are significantly lower than under that of auditory or audiovisual stimuli, they remain reliably above zero, suggesting that visual stimulation can augment auditory-evoked ASMR (Maeno and Kajimura, 2022). Other studies have shown that audiovisual observation of others’ actions elicits stronger tingling sensations than auditory perception of the same actions alone (Tada et al., 2021; Hozaki et al., 2025). Moreover, perceiving others’ actions that are congruent across visual and auditory modalities increases the likelihood and intensity of ASMR compared with incongruent actions (Maeno and Kajimura, 2022; Jones et al., 2025). Collectively, these findings suggest that auditory and visual information interact to enhance ASMR experiences.

One visual characteristic that may further modulate auditory-evoked ASMR is perspective. Actions that produce ASMR-eliciting sounds can be presented from either a first-person or a third-person perspective. In typical ASMR videos available on the internet, the actor (ASMRtist) often directs sounds toward a dummy head or microphone. The perception of such scenes can be considered a third-person perspective, in which the viewer observes the actor’s actions. By contrast, in the first-person perspective, the actor produces sounds while attending to the participant, directing the actions toward them rather than toward a recording device. Consequently, the actor appears more proximate and personally engaged with the participant in the first-person perspective. To what extent, then, does this difference in perspective influence the audiovisual interaction underlying the ASMR experience?

Another line of research has suggested that visual perspective modulates multisensory perception. For example, the rubber hand illusion (Botvinick and Cohen, 1998) is stronger when the rubber hand is presented from the first-person perspective than from the third-person perspective (Petkova et al., 2011; Maselli and Slater, 2013; Carey et al., 2019). Moreover, studies of the rubber hand illusion and social touch have shown that first-person embodiment enhances emotional responses to tactile stimulation on the embodied limb (Fahey et al., 2019). Given that ASMR may reproduce the comforting effects of social bonding behaviors (Lochte et al., 2018; Poerio et al., 2018; Hozaki et al., 2025) such as being touched (Suvilehto et al., 2019), it is reasonable to expect that the first-person perspective would amplify ASMR-related sensations compared to the third-person perspective.

A recent study found ASMR ratings differ between audiovisual stimuli categorized according to whether the actor’s attention was directed toward a target in the frame or toward an implied viewer outside of the frame (Jones et al., 2025). However, because the content of stimulation differs between the two types of videos (e.g., typing on a keyboard vs. touching microphones with soft fluffy material), the difference in stimuli such as content, rather than perspective, may explain the difference. Thus, it remains unclear to what extent visual perspective modulates ASMR during audiovisual perception of others’ actions.

In the present study, we conducted two preregistered experiments to test the hypothesis that visual perspective influences the audiovisual perception of objects that evoke ASMR. Participants rated their ASMR intensity after viewing and listening to audiovisual stimuli depicting objects that produce characteristic ASMR sounds. Sound was matched across the task conditions. In Experiment 1, participants viewed the stimuli from the third-person perspective. In Experiment 2, we directly compared the first- and third-person perspectives by using a head mount display (HMD). To examine the influence of visual stimulation on audition, we manipulated the spatiotemporal congruency of the stimuli. Based on prior studies (Hozaki et al., 2025; Maeno and Kajimura, 2022; Jones et al., 2025), we predicted that congruent audiovisual presentations would elicit stronger ASMR sensations than incongruent ones, regardless of the perspective. Furthermore, drawing on findings from body-ownership research showing stronger illusions from the first-person perspective, we predicted that multisensory enhancement of ASMR would be more pronounced in the first-person than in the third-person perspective.

## Experiment 1

### Methods

#### Participants

Forty-six naïve volunteers (20 males and 26 females; mean age = 19.7 years, range = 18–33) participated in this study. The study protocol was approved by Research Ethics Committee, Graduate School of Intercultural Studies, Kobe University (approval number: 2023-4). Written informed consent was obtained from all participants prior to participation. All procedures were conducted in accordance with the approved ethical guidelines. We estimated the effect size from previous studies (Tada et al., 2021; Maeno and Kajimura, 2022) and required sample size using G*Power 3.1.9.7 (Faul et al., 2007). With *α* = 0.05 and power (1–β) = 0.8, a minimum of 41 participants was needed to detect a medium effect size (*d* = 0.4) for one-tailed *t* tests. Thus, we recruited more than the minimum number of participants in the case that some data cannot be used for analyses due to technical problems. A pre-screening questionnaire assessed prior ASMR experience and confirmed that all the participants experience ASMR before.

#### Experimental design and stimuli

The design of the experiment was preregistered at OSF.^1^ The purpose of this experiment is twofold. First, we hypothesized that ASMR intensity should be lower in videos where a temporal mismatch is introduced between auditory and visual stimuli (asynchronous condition) compared to videos where both are perfectly synchronized (synchronous condition). Second, ASMR intensity should be higher in conditions when any visual information (e.g., image) related to the source of sound is visually presented than no such information was provided. For this purpose, the experiment involves the task with one within-subject factor (visual condition) and dependent variables were ASMR intensity, valence, and arousal level. In addition, the participants answered questionnaires and participated in the supplementary task as shown below.

**Figure 1:**
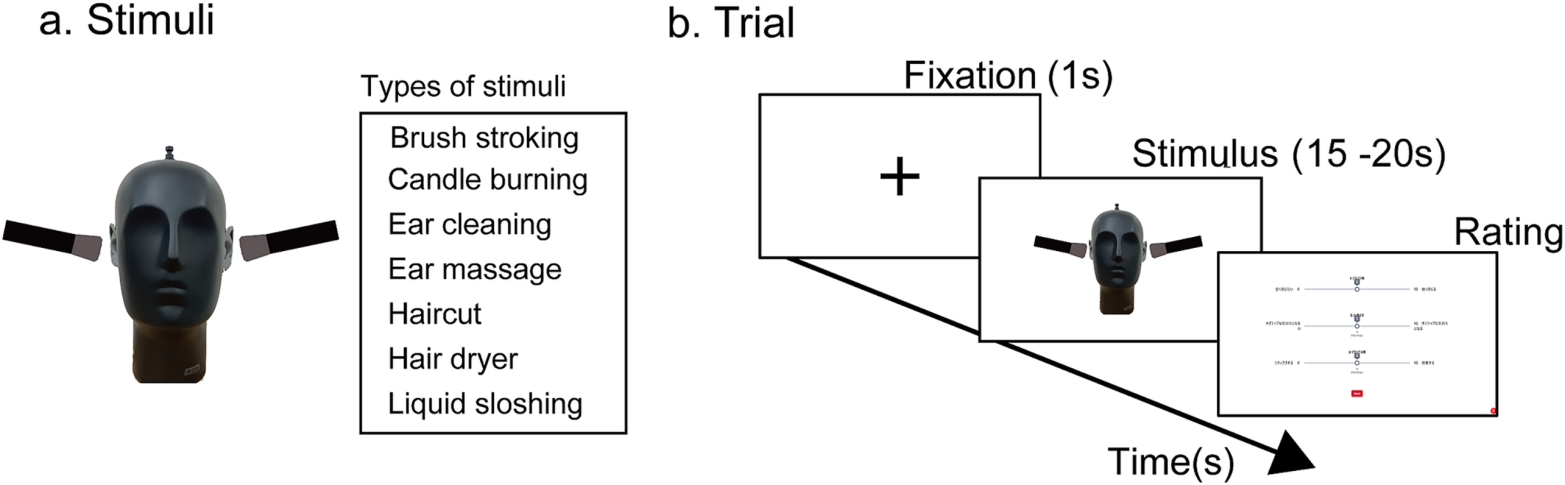
Experiment 1. **(a)** Stimuli. Seven types of audiovisual stimuli were prepared for the main experiment. In each stimulus, an experimenter generated sounds around a dummy head. The face of the person producing the sounds was not shown. The stimuli illustrated in the figure were conceptually based on a publicly available YouTube video (https://www.youtube.com/watch?v=ChKGbkLOokA) and were recreated by the authors using a dummy head and real objects to visualize the setup. **(b)** Trial sequence. After a 1 s fixation cross, a stimulus lasting 15–20 s was presented. Following stimulus presentation, participants used a visual analog scale (VAS) with their right hand to rate ASMR intensity, pleasantness, and arousal.

Stimuli were based on a publicly available YouTube video.^2^ Eight short segments (15–20 s) were selected from the video depicting brush stroking, candle burning, ear cleaning, ear massaging, hair cutting, hair drying, liquid sloshing, and bubble wrap tapping (Figure 1a). Each segment was trimmed so that the duration of a single clip ranged from 15 to 20 s. In these clips, a dummy head was presented facing forward while an actor manipulated objects to produce sounds around the head, with the actor’s face not visible. The clip duration was determined based on a previous study showing that a 20-s observation period was sufficient to elicit ASMR (Maeno and Kajimura, 2022). One segment (bubble wrap tapping) was used for practice, and the remaining seven were used in the main trials.

We created the four types of video clips from each segment: video clips for Synchronous condition, Asynchronous condition, Static Image condition, and Mosaic Image condition. The auditory stimuli were identical between these conditions. No audio processing (e.g., noise reduction or filtering) was applied to stimuli. We edited visual stimuli with video editing software (PowerDirector 365, CyberLink Corp., New Taipei City, Taiwan) in the following procedures. First, the original segment, of which visual and auditory information are already temporally aligned, was used for the Synchronous condition. Second, we created stimuli for the Asynchronous condition by shifting the timing of the visual and auditory stimuli. Specifically, a temporally offset portion of the visual stimulus was reassigned within the clip to introduce a temporal lag between audition and vision while preserving the overall stimulus content. The audiovisual timing lag (2–5 s) was determined based on pilot testing to ensure that audiovisual incongruence was clearly perceived. Because the stimuli involved directional motion detectable in both audition and vision, the lag duration was selected so that auditory and visual motion directions were mismatched.

For the Static image condition, we made stimuli in which a still image of visual stimulus was presented with the original audio clip. The middle frame of the segment was used as the static image. Finally, stimuli in the Mosaic Image condition were the same as the stimuli for the Static image condition except that the image was mosaic-filtered. Collectively, 28 stimuli (4 conditions × 7 video segments for the main experiment) were prepared for the experiment.

#### Apparatus

The experiment was implemented using the Gorilla Experiment Builder^3^ only accessible to the participants and experimenters. Stimuli were presented on a 23.8-inch LCD monitor (S2421HS; Dell Inc., Round Rock, TX, USA; refresh rate: 60 Hz; spatial resolution: 1,920 × 1,080 pixels) controlled by a desktop PC (DAIV; Mouse Computer Co., Ltd., Tokyo, Japan). The temporal precision of consumer-grade LCD monitors used for visual stimulation has been empirically characterized in previous work (Zhang et al., 2018). Participants sat at a fixed viewing distance of 41 cm from the screen, maintained using a chinrest (TKD-UK1; NAMOTO Co., Ltd., Matsudo, Japan). Auditory stimuli were delivered through headphones (ATH-M40x; Audio-Technica Corp., Tokyo, Japan) connected to a sound card (Sound BlasterX AE-5; Creative Technology Ltd., Singapore). The experiment was conducted in a quiet room. The visual stimuli, corresponding to the maximum range of the objects and ASMRtist’s movements, subtended a visual angle of 24.9° (vertical) × 45.8° (horizontal). The sound level was adjusted individually so that participants could comfortably listen to the audio stimuli.

#### The main task and procedure

In each trial, participants viewed a stimulus and then rated ASMR intensity, valence (pleasantness), and arousal on 0–10 visual analog scales (VAS; Figure 1b). Specifically, for ASMR intensity, participants rated the maximum tingling sensation they experienced from “not at all” (0) to “extremely strong” (10). For valence, they rated the maximum pleasantness they felt from “negative feelings” (0) to “positive feelings” (10). For arousal, participants rated their level of excitement from “relaxed” (0) to “excited” (10). Four stimuli from each condition were presented as practice trials, after which each stimulus was presented twice. The order of stimulus presentation was pseudo-randomized across participants.

#### The supplementary task

After the main experiment, participants completed a supplementary task designed to assess their ability to detect asynchrony between auditory and visual stimuli. The task was identical to the main experiment, except those participants rated the perceived degree of synchrony on a scale from “not synchronized at all” (0) to “very synchronous” (10).

#### Questionnaires

To examine the effects of individual traits on ASMR experiences, we administered the following questionnaires.

##### ASMR questionnaire

ASMR-related experiences were assessed using the ASMR Questionnaire (Barratt and Davis, 2015; Tada et al., 2022). Only participants who had previously watched ASMR videos and experienced ASMR-related tingling sensations completed this measure. Participants rated eight statements describing mental and physical states during the tingling experience (e.g., “completely focused on what I am watching,” “things seem to happen automatically”). Higher scores indicate greater tingling intensity, immersion, and a flow-like state (Csikszentmihalyi, 1990).

##### Autism Spectrum quotient (AQ)

Previous studies have examined the relationship between ASMR experiences and autistic traits (Barratt and Davis, 2015; Tada et al., 2022). Autistic traits were assessed using the Autism Spectrum Quotient (AQ; Baron-Cohen et al., 2001). A total AQ score was calculated for each participant, with higher scores indicating stronger autistic traits.

##### Adult/adolescent sensory profile (AASP)

Previous studies have also explored the relationship between ASMR experiences and individual sensory differences (Poerio et al., 2022, 2023). Here, individual differences in sensory processing were assessed using the Adult/Adolescent Sensory Profile (AASP; Brown and Dunn, 2002). This questionnaire yields scores across four dimensions: low registration, sensation seeking, sensory sensitivity, and sensation avoiding.

#### Data analyses

Data were analyzed using Microsoft Excel and the R package^4^ with the ANOVAKun toolbox (version 4.8.9; https://riseki.cloudfree.jp/?ANOVA%E5%90%9B). Following the preregistration, we conducted confirmatory analyses (Wagenmakers et al., 2012) in which *a priori* hypotheses were tested using one-tailed paired *t*-tests without correction for multiple comparisons (Kirk, 2013; Rubin, 2021; Hales, 2024). This approach is analogous to the use of linear contrasts commonly applied in fMRI analyses (Friston et al., 2007) and was adopted to test the preregistered a priori hypotheses (Atilgan et al., 2023). To test the first hypothesis, ASMR intensity, valence, and arousal ratings were compared between the Synchronous and Asynchronous conditions. To test the second hypothesis, ratings for the Mosaic Image condition were compared with those for each of the other conditions. Subsequently, *post hoc* exploratory analyses were conducted using one-way repeated-measures ANOVAs with the factor of visual condition (Synchronous, Asynchronous, Static Image, and Mosaic Image) on each dependent variable (i.e., ASMR intensity, valence, and arousal ratings in the main task, and synchrony ratings in the supplementary task), followed by two-tailed paired t tests with Shaffer’s correction. Greenhouse–Geisser corrections were applied to degree of freedom when sphericity assumption was violated. Finally, we conducted correlation analyses between the scores on each questionnaire (ASMR score, AQ, and AASP) and the synchronization effect (synchronized condition vs. asynchronous condition) to examine their relationships.

### Results

#### ASMR intensity

**Figure 2:**
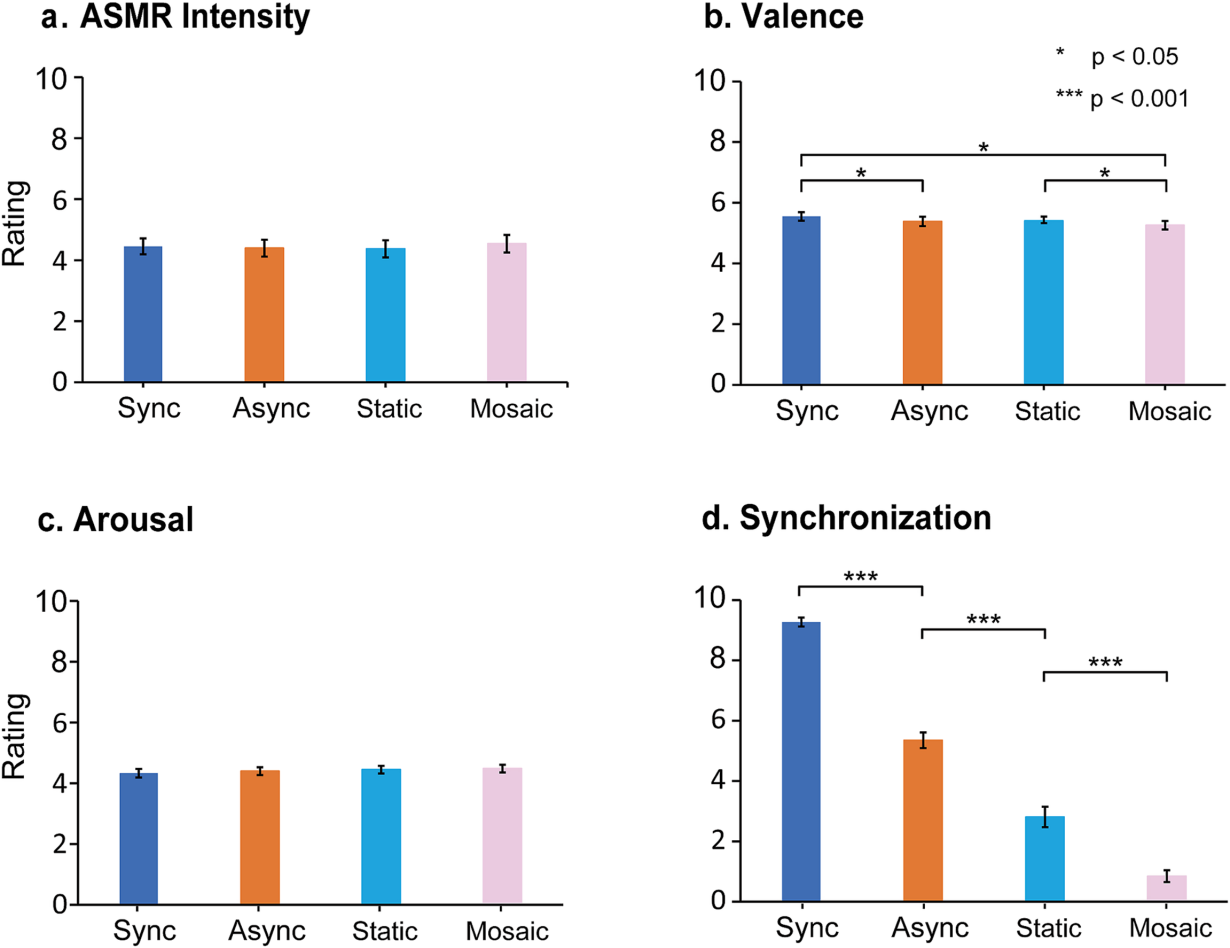
Ratings of ASMR intensity **(a)**, valence **(b)**, arousal **(c)**, and synchronization (d) are shown. Data represent the mean ± standard error of the mean (SEM) across 46 participants. Sync, Async, Static, and Mosaic indicate the synchronous, asynchronous, static image, and mosaic image conditions, respectively.

Figure 2a shows the ASMR intensity results. We first conducted a confirmatory analysis to test the preregistered hypotheses. A one-tailed paired *t*-test revealed no significant difference between the Synchronous and Asynchronous conditions (*p-*uncorrected = 0.3, *d* = 0.07). Similarly, one-tailed *t*-tests showed that the Mosaic Image condition did not significantly differ from any of the other conditions (*p-*uncorrected values > 0.71, absolute d values < 0.20). We then conducted a posteriori analysis by conducting a one-way repeated-measures ANOVA with four visual conditions (Synchronous, Asynchronous, Static Image and Mosaic Image). This analysis revealed no significant effect (*p* = 0.63, η_p_^2^ = 0.01). Finally, as a supplementary analysis, we examined ASMR occurrence rate by calculating the frequency of non-zero ASMR intensity values. Across all conditions, ASMR occurrence rates were high (87.3 ± 3.12%, mean ± SEM). The same one-way repeated-measures ANOVA on the occurrence rate revealed no significant main effect (*p* = 0.43, η_p_^2^ = 0.02).

#### Valence

Figure 2b shows the pleasantness ratings. Confirmatory one-tailed paired t tests showed that the Synchronous condition was rated significantly more pleasant than the Asynchronous condition [t(45) = 2.36, *p-*uncorrected = 0.011, d = 0.35] and the Mosaic Image condition [t(45) = 2.97, *p-*uncorrected = 0.002, d = 0.44]. The Static Image condition was rated significantly more pleasant than the Mosaic condition [t(45) = 1.85, *p-*uncorrected = 0.036, d = 0.15]. There was no significant difference between the Asynchronous and Mosaic Image conditions (*p-*uncorrected value = 0.095, d = 0.20). As an exploratory analysis, a one-way repeated-measures ANOVA with the four visual conditions on pleasantness ratings revealed a significant main effect of condition [*F*(2.48, 111.38) = 4.13, *p* = 0.012, η_p_^2^ = 0.08]. *Post-hoc* paired *t* tests using Shaffer’s correction confirmed a significant difference between the Synchronous condition and the Mosaic condition [*p-*corrected = 0.028, d = 0.30].

#### Arousal

Figure 2c shows the results for arousal ratings. Confirmatory one-tailed paired t-tests revealed no significant differences between any of the conditions (*p-*uncorrected values > 0.6, absolute d values < 0.20). A one-way repeated-measures ANOVA with four visual conditions (Synchronous, Asynchronous, Static Image, and Mosaic Image) also showed no significant main effect (*p* = 0.47, η_p_^2^ = 0.02).

#### Perceived audiovisual synchrony

Figure 2d shows the pattern of perceived audiovisual synchrony ratings in the supplementary task. Because this task was not preregistered, we performed a one-way repeated-measures ANOVA (with four visual conditions) on perceived synchrony. This test revealed a significant main effect [*F*(2.51, 112.73) = 280.59, *p* < 0.001, η_p_^2^ = 0.86]. Post-hoc pairwise comparisons (pared t tests with Shaffer’s correction) showed significant differences between all pairs of the conditions with the synchronous condition rated as most temporally aligned, followed by Asynchronous, Static Image, and Mosaic Image conditions (*p-*corrected values < 0.001, absolute d values > 1.18).

#### Correlation between synchrony effects and questionnaire scores

Finally, as a non-preregistered exploratory analysis, we examined if scores of questionnaires (ASMR questionnaire score, AQ total score, and scores for four quadrants in AASP) are related to the synchrony effect, revealed by the Synchronous vs. Asynchronous condition, in the main experiment. However, none of the questionnaire’s scores significantly predicted the synchrony effects for ASMR intensity, valence, and arousal (absolute *r* values < 0.3).

Collectively, the results of Experiment 1 only partially supported our *a priori* hypothesis. Specifically, valence ratings showed that the Synchronous condition was perceived as significantly more pleasant than the Asynchronous and Mosaic Image conditions. We then examined the effect of visual perspective on ASMR in Experiment 2, in which the visual stimuli were presented through an HMD that manipulated the observer’s perspective.

## Experiment 2

### Methods

#### Participants

Forty-two volunteers (15 males and 27 females; mean age = 21.7 years, range = 18–30) participated in the experiment. The study protocol was approved by Research Ethics Committee, Graduate School of Intercultural Studies, Kobe University (approval number: 2023-4). Written informed consent was obtained from all participants prior to participation. We used the same sample size estimation as Experiment 1. The design of the experiment was preregistered at OSF.^5^ Participants were not prescreened. Twenty-seven volunteers (64%) reported that they had watched ASMR videos and experienced ASMR before this experiment, whereas the remaining 15 participants had never watched ASMR videos or had never experienced ASMR while watching such videos. Eight volunteers had also participated in Experiment 1.

#### Experimental design

The purpose of this experiment was to examine the effect of visual perspective on sound-induced ASMR experiences. We manipulated two within-subject factors: synchrony (synchronous and asynchronous conditions) and visual perspective (first-person and third-person perspectives). We preregistered the following hypotheses. First, we hypothesized both an interaction between visual perspective and synchrony and effects of each factor such that ASMR intensity and emotional valence would be highest, and arousal lowest, in the first-person synchronous condition (a supra-additive effect). Second, we hypothesized a significant correlation between ASMR Questionnaire scores and the sensation seeking subscale of the AASP, following previous research (Poerio et al., 2023).

#### Stimuli

**Figure 3:**
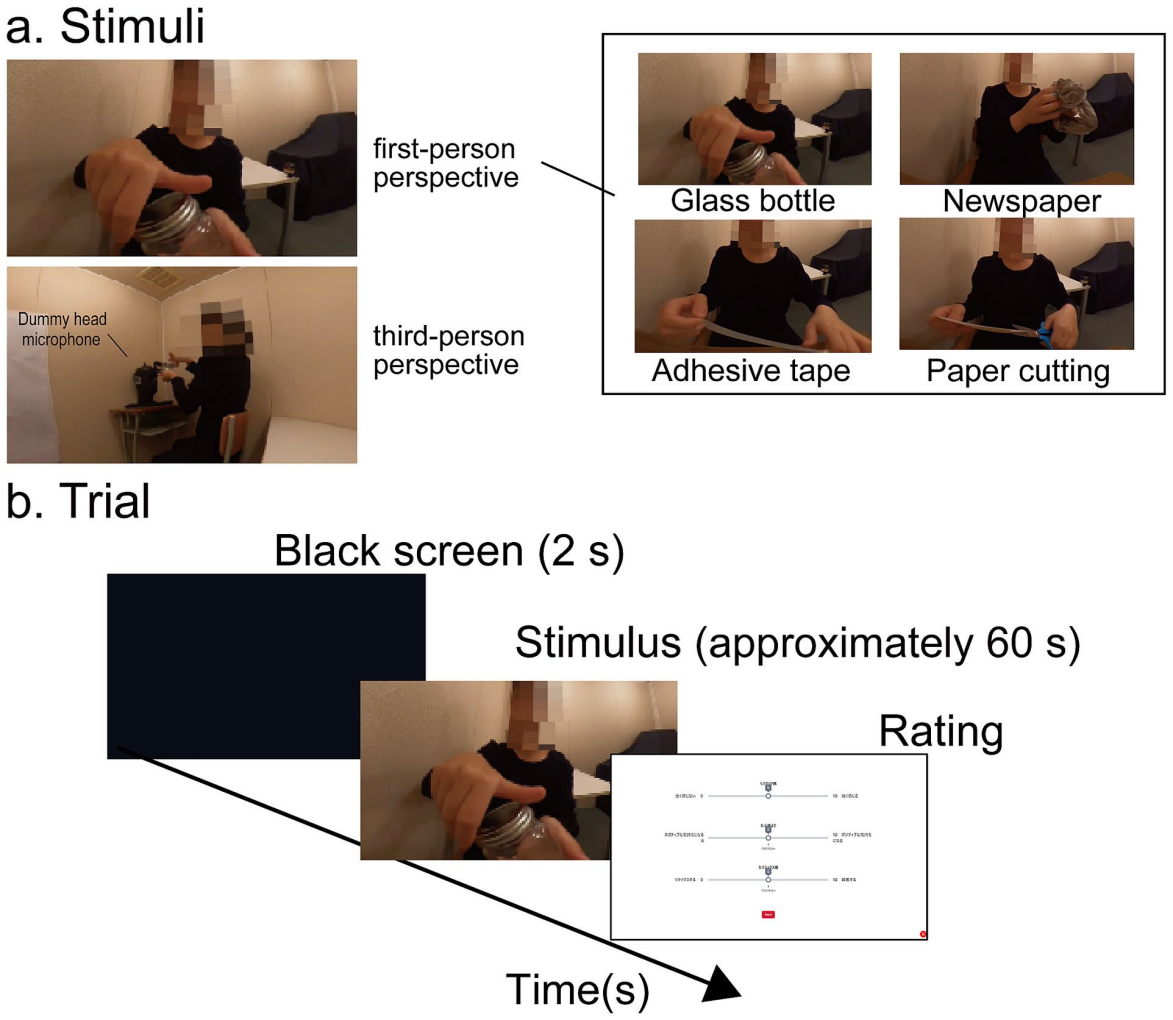
Experiment 2. (**a**) Stimuli. Participants performed the task using an HMD to examine the effect of visual perspective. Examples of 3D camera images projected onto a 2D screen are shown. **(b)** Trial sequence. Each trial began with the presentation of a stimulus video, after which participants rated ASMR intensity, valence, and arousal.

Audiovisual 3D stimuli were recorded in a soundproof room using a binaural dummy-head microphone (B1-E; Binaural Enthusiast, Chula Vista, CA, USA) and two 360-degree cameras (Max; GoPro Inc., San Mateo, CA, USA). One camera was mounted on top of the dummy head and captured the experimenter (S.N) producing sounds with various objects. Because this view shows a situation in which the experimenter was performing the actions toward the participants, this condition was termed the first-person perspective (1PP) condition. The other camera recorded the same actions from a table placed to the left of the experimenter. This view shows a situation in which a participant observes the experimenter producing sounds directed toward another person (the dummy head), similar to Experiment 1. A slanted camera angle was chosen to allow clear observation of the experimenter’s actions without her body occluding the manipulated objects. This setup is similar to a procedure used for body ownership illusions (Petkova et al., 2011). We created four types of stimuli for the main experiment (Figure 3a right): Opening and closing a glass bottle (Glass bottle), Crumpling a newspaper (Newspaper), Unroll some adhesive tape (Adhesive tape), and Cutting paper (Paper cutting). An additional stimulus mimicking scissor cutting was used for practice. Each audiovisual clip lasted approximately 60 s. The clothes of the experimenter and background were identical across the stimuli.

Audio and video recordings were edited using PowerDirector 365 (CyberLink Corp., New Taipei City, Taiwan) to create stimuli for the four experimental conditions: 1PP-synchronous, 1PP-asynchronous, 3PP-synchronous, and 3PP-asynchronous. The experimenter’s actions were initially recorded for approximately 120–180 s. From these recordings, 10–15 s video segments were extracted from two 360° cameras and temporally aligned with audio captured using a dummy head microphone. The aligned 1PP and 3PP video segments were used for stimuli in the 1PP-synchronous and 3PP-synchronous conditions, respectively. For the asynchronous conditions, the same recordings as those used in the synchronous conditions were employed as stimuli; however, the audio track was shifted backward in time relative to the corresponding video (by 4.2–7.0 s) to maximize temporal misalignment, while the audio component remained identical to that used in the synchronous condition. The timing lag was determined using the same criteria as in Experiment 1; however, in Experiment 2 the manipulation consisted solely of a temporal shift, without reassignment of visual segments.

All stimuli across the four conditions had a fixed duration of approximately 60 s, created by looping the 10–15-s segments. Mosaic filtering was applied to obscure the experimenter’s face, and no audio post-processing (e.g., filtering or noise reduction) was applied. A total of 16 clips were produced (4 conditions × 4 sound sources). The visual stimuli (maximum range of the experimenter’s actions and object motion) were presented at a visual angle of 96.0° (vertical) × 103.8° (horizontal) in the first-person perspective and 40.0° (vertical) × 83.1° (horizontal) in the third-person perspective. Sound volume was adjusted individually so that participants felt comfortable listening to the stimuli.

#### Apparatus

Stimuli were presented using an HMD (Quest 3, Meta, Palo Alto, CA, USA). A face cover (Face Cover for Quest 3, Maecker Co., Ltd., Tokyo, Japan) was attached to the headset to minimize unintended visual input (e.g., ambient room light). The HMD operated at a refresh rate of 72 Hz with a display resolution of 2,064 × 2,208 pixels per eye. A battery-powered strap (Meta, Palo Alto, CA, USA) was added to the HMD to stabilize the headset position and prevent power loss during the experiment. Visual stimuli were presented via a video playback application installed on the HMD (SKYBOX VR Video Player; SKYBOX Studio, Irvine, CA, USA). Because the HMD was not compatible with the over-ear headphones used in Experiment 1, in-ear headphones (E500, final Inc., Kawasaki, Japan) were used instead. The experiment was conducted in a quiet room.

#### Procedures

Participants received instructions about the experimental procedure and completed a written informed consent form. After fitting the HMD and headphones, they completed a short practice session using audiovisual stimuli to adjust the sound volume and familiarize themselves with the rating procedure. In the main task, 16 stimuli were presented across two blocks (8 clips per block) with a short break between blocks (Figure 3b). Each trial began with a 2-s black screen, followed by the audiovisual presentation of a video clip, and ended with a visual analogue scale (VAS; 0–10) for rating the maximum ASMR intensity, valence, and arousal experienced during the observation of the stimuli. Because manipulation of the VAS within the 3D display was difficult, participants verbalized their ratings, which were recorded by the experimenter.

#### Questionnaires

After completing the main task, participants answered a post-experimental questionnaire to assess whether they noticed audiovisual asynchrony in the asynchronous conditions. Because the ASMR stimuli were created in-house, we also examined which parts of the body participants experienced tingling sensations and whether their spatial extent was related to ASMR intensity. For this purpose, we used the emBODY tool (Suvilehto et al., 2019; Fukuoka et al., 2025), in which participants were asked to paint body maps indicating the areas where they felt tingling sensations. Finally, participants completed the ASMR Questionnaire and AASP to examine the relationship between ASMR experience score and sensation seeking score in the AASP, following a previous study (Poerio et al., 2023).

#### Analyses

The same software as in Experiment 1 was used for data analysis. As in Experiment 1, confirmatory analyses were conducted by calculating ratings in the synchronous condition relative to the asynchronous condition within each visual perspective (synchrony effect). We then computed the interaction term by subtracting the synchrony effect in the third-person perspective from that in the first-person perspective (e.g., Atilgan et al., 2023). The synchrony and interaction effects were statistically evaluated using one-tailed one-sample *t*-tests without correction for multiple comparisons. As a posteriori analyses, we performed repeated-measures ANOVAs on each VAS rating with within-subject factors of visual perspective (first-person and third-person) and audiovisual synchrony (synchronous and asynchronous) as independent variables.

For the body-map data, we used an in-house program in MATLAB (R2022b, Mathworks, Natick, MA, USA), following the procedures described in previous studies (Suvilehto et al., 2019; Fukuoka et al., 2025). The colored images were binarized so that the amount of time a participant spent coloring would not influence the results. Pixelwise mean intensity values were then computed to represent agreement in tingling sensations across participants. Finally, the total body area was calculated as the proportion of colored pixels within the body outline for each participant’s map.

## Result

### ASMR intensity

**Figure 4:**
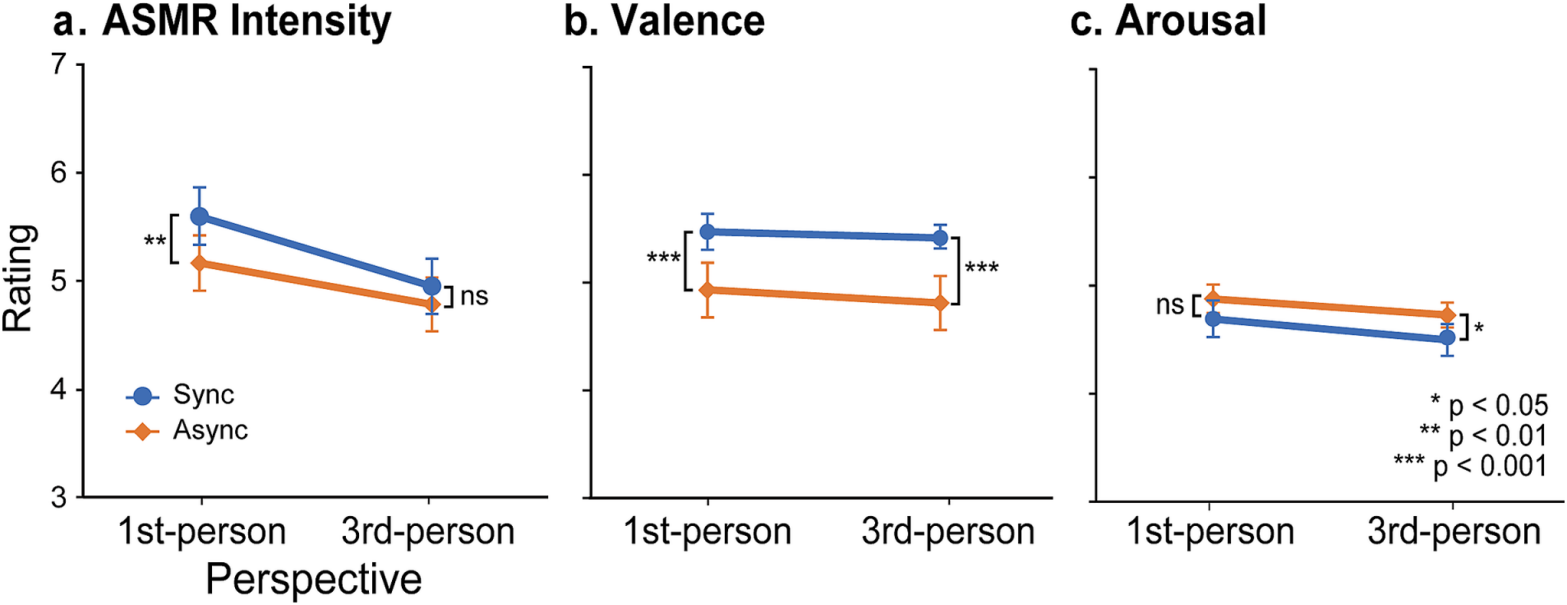
Results of experiment 2. Ratings of ASMR intensity **(a)**, valence **(b)**, and arousal **(c)** are shown. Error bars indicate SEM across 42 participants.

Figure 4a shows ASMR intensity in Experiment 2. Supplementary Tables 1, 2 provide the corresponding descriptive statistics (mean ± SEM). The overall pattern was consistent across stimulus types. As in Experiment 1, preregistered *a priori* hypotheses were evaluated using one-tailed paired *t*-tests (uncorrected for multiple comparisons). For first-person perspective videos, ASMR intensity was significantly higher in the synchronous condition (1PP-synchronous) than in the asynchronous condition (1PP-asynchronous) [t(41) = 2.78, *p-*uncorrected = 0.004, d = 0.43]. In contrast, no significant difference was found between conditions for third-person perspective videos (3PP-synchronous vs. 3PP-asynchronous, *p-*uncorrected = 0.087, d = 0.21). The same t-test on Synchrony effects (synchronous minus asynchronous) revealed that the synchrony effect was greater for the first-person perspective than for the third-person perspective, showing the two-way interaction effect [t(41) = 2.31, *p-*uncorrected = 0.013, d = 0.36].

We also examined whether ASMR intensity differed between visual perspectives regardless of synchrony. One-tailed paired t-tests showed higher ASMR intensity in the first-person than in the third-person perspective for both the synchronous condition [t(41) = 5.53, *p-*uncorrected < 0.001, d = 0.85], and the asynchronous condition [t(41) = 3.37, *p-*uncorrected < 0.001, d = 0.52].

As an exploratory analysis, we performed two-way repeated measures ANOVA [two levels of perspective × two levels of synchrony] with ASMR intensity as a dependent variable. This analysis revealed a significant main effect of perspective [*F*(1, 41) = 26.23, *p* < 0.001, η_p_^2^ = 0.39], and a significant main effect of synchrony [F(1, 41) = 5.52, *p* = 0.024, η_p_^2^ = 0.12]. The interaction between perspective and synchrony was significant [F(1, 41) = 5.35, *p* = 0.026, η_p_^2^ = 0.12]. These results are consistent with the results of the confirmatory analyses.

Finally, as in Experiment 1, we conducted a supplementary analysis on ASMR occurrence rate by examining the frequency of ASMR intensity values greater than zero. Across all conditions, ASMR occurrence rates were high (95.0 ± 0.02%). The same two-way repeated-measures ANOVA on the occurrence rate revealed no significant main effects or interaction (all *p* values > 0.1, η_p_^2^ < 0.05).

### Valence

Figure 4b shows the valence ratings. The overall pattern was consistent across stimulus types (Supplementary Tables 1, 2). As in the ASMR intensity, we conducted confirmatory analyses. Pleasantness ratings were significantly higher in the synchronous condition compared to the asynchronous condition for the first-person perspective [t(41) = 4.07, *p-*uncorrected < 0.001, d = 0.63] and for the third-person perspective conditions [t(41) = 4.22, *p-*uncorrected < 0.001, d = 0.65]. We then examined the interaction effect by conducting paired *t* tests on the synchrony effect between first and third person perspectives. However, this test revealed no significant effect (*p-*uncorrected = 0.72, absolute d = 0.16). We also compared first- and third- person perspectives in each level of synchrony. No significant differences were found, regardless of whether the audiovisual stimuli were synchronous (*p-*uncorrected = 0.36, absolute d = 0.06) or asynchronous (*p-*uncorrected = 0.16, absolute d = 0.15). Finally, an exploratory two-way repeated-measures ANOVA (2 perspectives × 2 synchrony) revealed a significant main effect of synchrony [*F*(1, 41) = 21.95, *p* < 0.001, η_p_^2^ = 0.35]. Neither the main effect of perspective nor the interaction was significant (*p* values > 0.4, η_p_^2^ < 0.02).

### Arousal

Figure 4c shows the arousal ratings. The overall pattern was again consistent across stimulus types (Supplementary Tables 1, 2). One-tailed paired t-tests to test preregistered hypotheses showed a significantly greater arousal level for the asynchronous than the synchronous condition for the third person perspective [t(41) = −2.21, *p-*uncorrected = 0.016, d = −0.34], but only a trend toward significance for the first-person perspective [t(41) = −1.54, *p-*uncorrected = 0.066, d = −0.24]. The synchrony effect (synchronous – asynchronous rating) showed no significant difference between the two perspective conditions, indicating no significant two-way interaction (*p-*uncorrected = 0.63, absolute d = 0.05). Finally, we compared the ratings between the two perspectives for each level of synchrony. One-tailed paired t tests showed no significant differences between the two perspectives under the synchronous condition or under the asynchronous condition (*p-*uncorrected values > 0.8, absolute d values < 0.22).

A posteriori 2 × 2 repeated measures ANOVA (2 levels of perspective × 2 levels of synchrony) showed a significant main effect of synchrony [F(1, 41) = 5.39, *p* = 0.025, η_p_^2^ = 0.12]. However, neither the main effect of perspective nor its interaction effect was significant (*p* values > 0.10, η_p_^2^ values < 0.06).

### Post-experimental questionnaire

**Figure 5:**
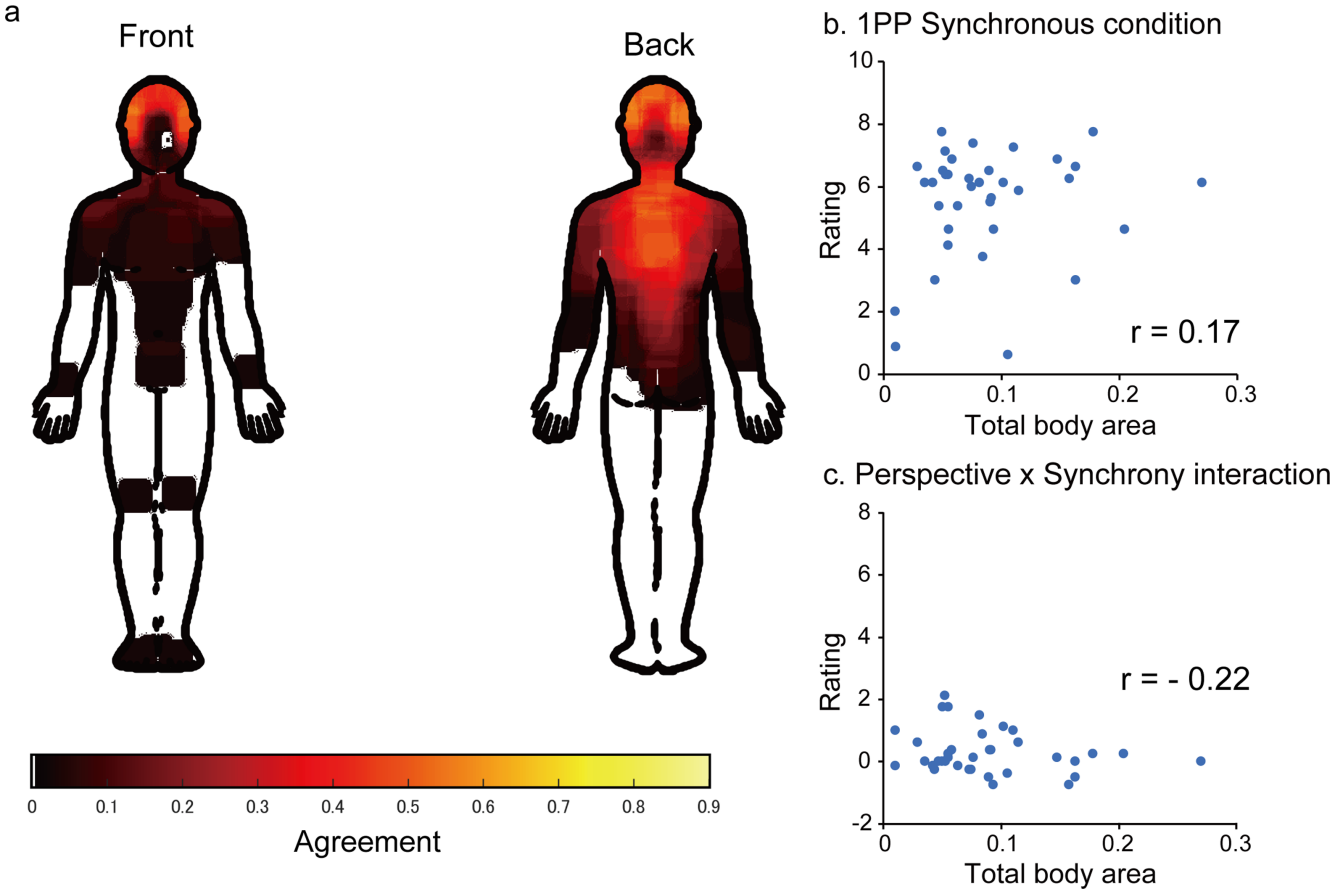
Body area where the participants felt tingling sensation and its relationship with the ASMR intensity (*n* = 36). **(a)** Agreement of body area where the participant felt tingling sensation in response to audiovisual stimulation (*n* = 36). White area indicates zero agreement. **(b,c)** Total body area (ratio of painted area relative to the whole body area) did not significantly predict the ASMR intensity in the first-person perspective (1PP) synchronous condition nor the perspective *x* synchrony interaction term.

Thirty-eight of the forty-two participants (90%) reported that they detected asynchrony in the stimulus presentation. As the ASMR stimuli were created in-house, we further characterized the topographical distribution of tingling sensations and examined whether their spatial extent was related to ASMR intensity. Figure 5a illustrates the body areas where participants perceived tingling sensations during the experiment. Most participants reported tingling in the upper body, particularly around the head and back, consistent with the topographical patterns of tingling sensations reported in previous studies (Barratt and Davis, 2015; Koumura et al., 2021; Jones et al., 2025). However, the spatial extent of tingling sensations (total body area), whether analyzed separately for the front and back of the body or combined across both sides, did not predict ASMR intensity in the first-person synchronous condition or the perspective × synchrony interaction effect (absolute *r* values < 0.31; Figures 5b,c).

### Comparisons between individuals with and without ASMR experience

As a non-preregistered exploratory analysis, we compared synchrony × perspective interaction effects between those who have experienced ASMR before (ASMR experiencers, *n* = 27) and those who have not experienced ASMR (non-ASMR experiencers, *n* = 15). Table 1 shows the descriptive results for each group. Welch’s two-sample *t* tests (with Shaffer’s correction over 3 pairs of comparisons) showed no significant differences (*p-*corrected values > 0.22, absolute d values < 0.18). In addition, Welch’s two-sample *t* tests on AASP scores (with Shaffer’s correction over 4 pairs of comparisons) showed that ASMR experiencers had greater sensation sensitivity scores in AASP than other scores [t(34.0) = 2.85, *p-*corrected = 0.028, d = 0.89].

**Table 1:** Descriptive statistics of sensory profiles and interaction effects of ASMR ratings in experiment 2.

Statistic	AASP				Interaction effects of ASMR ratings		
	Low registration	Sensation seeking	Sensory sensitivity	Sensation avoiding	ASMR intensity	Valence	Arousal
ASMR experiencers (*n* = 27)							
Mean	36.0	46.5	44.1*	39.7	0.38	0.12	−0.01
SEM	1.8	1.5	1.7	1.9	0.16	0.12	0.17
ASMR non-experiencers (*n* = 15)							
Mean	35.5	42.1	37.1	41.2	0.06	−0.43	0.15
SEM	1.8	1.5	1.8	1.9	0.11	0.28	0.23

*indicates significant group difference (p corrected for multiple comparisons over 4 quadrant scores of AASP).

### ASMR questionnaire and sensory profiles AASP

Finally, we tested our third a prior hypothesis in the preregistration; the correlation between ASMR questionnaire score (Barratt and Davis, 2015) and sensation seeking scores in AASP (Brown and Dunn, 2002). However, no significant correlation was observed (*r* = − 0.04). Likewise, exploratory analyses showed no significant correlation between ASMR questionnaire score and other scores in AASP was observed (absolute r values < 0.3).

## Discussion

The present study examined how audiovisual synchrony and visual perspective influence the experience of ASMR. In Experiment 1 that employed a third-person perspective, no effect of audiovisual synchrony effect on ASMR intensity was observed. By contrast, Experiment 2 revealed that ASMR intensity was significantly higher in the synchronous than in the asynchronous condition when the stimuli were presented from a first-person perspective, whereas this effect was absent for the third-person perspective. Valence ratings were largely higher in the synchronous condition than other conditions, regardless of perspective. For arousal, participants in the third-person condition reported feeling more relaxed in the synchronous than in the asynchronous condition, while no such effect was observed in the first-person condition.

### The effect of audiovisual synchrony and visual perspective on ASMR intensity

The main finding of the present study is that the effect of audiovisual synchrony was observed only in the first-person perspective condition. Previous studies have shown that visual information associated with sound sources can enhance ASMR responses (Tada et al., 2021; Maeno and Kajimura, 2022; Jones et al., 2025). The present study extends these findings by demonstrating that visual perspective modulates the audiovisual interaction underlying ASMR intensity. In the present study, audiovisual synchrony was examined using different methods of visual presentations in Experiments 1 and 2. Because visual and auditory stimuli were largely matched between the synchronous and asynchronous conditions in each experiment, the observed interaction between synchrony and perspective is unlikely to be explained by differences in low-level visual features (e.g., luminance and auditory intensity). In both experiments, the asynchronous condition involved a temporal offset of more than 2 s between the auditory and visual stimuli, and most of the participants detected this desynchronization during manipulation checks. Thus, it is unlikely that participants failed to perceive the temporal offset. Rather, the finding suggests that the effect of audiovisual synchrony on ASMR intensity is enhanced when the stimulation is experienced from a self-related, first-person perspective.

Why, then, was ASMR intensity modulated only by the first-person perspective? One possible explanation involves multisensory interaction processes closely linked to bodily self-representation. Studies on the rubber hand illusion (Botvinick and Cohen, 1998) have shown that the illusion is stronger when the rubber hand is placed in a first-person rather than a third-person orientation (Petkova et al., 2011; Maselli and Slater, 2013; Carey et al., 2019). For instance, Petkova et al. (2011) examined the strength of the full-body ownership illusion under conditions in which participants observed an artificial body from either the first- or third-person perspective. They found that the first-person visual perspective not only strengthened the subjective sense of body ownership but also enhanced the threat-evoked increase in skin conductance responses. Similarly, observing a rubber hand that is embodied as one’s own being stroked by an experimenter can evoke tactile pleasantness (Fahey et al., 2019). Given the framework that ASMR may reproduce the comforting effects of social bonding behaviors (Hozaki et al., 2025), it is possible that audiovisual stimulation in the first-person perspective amplifies ASMR-related sensations because participants may perceive themselves as the target of another’s action. Such first-person simulation may increase the anticipation of tactile stimulation to the head or neck through facilitated audiovisual interactions.

In the present study, the difference between the first-person and third-person perspectives inevitably introduced a difference in the perceived distance from the participants. Because the experimenter generated sounds close to the participants in the first-person perspective condition, the perceived proximity to the experimenter was greater than in the third-person perspective condition. Previous studies have examined how interaural acoustic characteristics influence ASMR experiences, such as interaural level difference (Koumura et al., 2021) and interaural cross-correlation (Shimokura, 2022). These findings suggest that the proximity of sound to the listener modulates ASMR intensity. In the context of audiovisual interactions, Liu and Kondo (2025) measured tingling intensity through real-time ratings during ASMR videos and found that the “Mic” category (direct contact with microphones) elicited the strongest tingling sensations, followed by other categories. Similarly, an exploratory analysis by Jones et al. (2025) showed that ASMR intensity and frequency increased in videos where the actor’s attention was directed toward an implied viewer outside the frame, compared with when attention was directed toward a target (e.g., a person or microphone) within the frame. Together, our results in conjunction with these findings suggest that the effect of first-person visual perspective on audiovisual interactions may partly reflect differences in perceived proximity.

By contrast, we examined the effect of audiovisual synchrony in the third-person perspective under the two different conditions. More specifically, in Experiment 1, participants viewed a 2D image of the dummy head facing the experimenter, whereas in Experiment 2, they viewed a 3D image of the dummy head from an oblique perspective. Regardless of visual proximity cues (e.g., viewing angle, orientation, or apparent distance) between the experiments, neither experiment showed a synchrony effect on ASMR intensity in the third-person condition. In contrast, a synchrony effect emerged in the first-person perspective. This pattern suggests that the influence of proximity on audiovisual synchrony may be more limited in the third-person perspective than in the first-person perspective.

Previous studies have reported congruency effects on ASMR occurrence (Maeno and Kajimura, 2022) and perceived tingles (Jones et al., 2025), even when a third-person perspective was explicitly employed (Jones et al., 2025). One possible explanation lies in differences in how audiovisual incongruence was implemented. In Maeno and Kajimura (2022), incongruent stimuli were created by scrambling audiovisual pairings, whereas Jones et al. (2025) temporally segmented and desynchronized the stimuli. In contrast, the present study introduced incongruence by shifting the timing between auditory and visual stimuli while largely preserving overall action structure and audiovisual content. This more stringent manipulation may have reduced sensitivity to congruency effects on ASMR intensity in the third-person perspective.

### The effect of audiovisual synchrony and visual perspective on valence ratings

Valence ratings tended to be higher in the synchronous condition than in the other conditions. In Experiment 1, the synchronous condition was rated as more pleasant than both the asynchronous condition and the auditory only condition with mosaic images (Mosaic Image condition). In Experiment 2, the synchrony effect, reflected in higher valence ratings for the synchronous compared with the asynchronous condition, was observed regardless of visual perspective. Together, these results suggest that sound-induced positive emotion is enhanced by the presence of synchronously presented visual stimulation.

Our initial prediction was that, because ASMR effect is associated with a positive emotion, an increase of ASMR leads to the increase of pleasantness ratings. However, patterns of pleasantness ratings were not identical to patterns of ASMR intensity; the former was more sensitive than the latter to the difference between synchronous condition and others. There are two accounts for this finding. First, the mechanism underlying a positive emotion triggered by audiovisual stimulation differs from the mechanism underlying the induction of ASMR intensity. For instance, since visual stimulation in our study is associated with actors’ actions near own or other’s head, visual observation of actions may be sufficient to induce emotions possibly via action-observation system (Bastiaansen et al., 2009; Kitada et al., 2010, 2013). This view is consistent with other studies in which watching others are tactually stimulated could activate the somatosensory cortex (Blakemore et al., 2005) and the limbic system (Singer et al., 2004). Second, asynchronous condition could add more cognitive effort to associate visual and auditory stimulation, and such cognitive effort for binding audiovisual stimulation for rating may be reflected in relatively lower pleasantness ratings than synchronous condition.

### The effect of audiovisual synchrony and visual perspective on arousal ratings

Like valence ratings, arousal ratings were comparable between the two perspective conditions. Arousal levels were significantly lower in the synchronous condition than in the asynchronous condition for the third-person perspective, and a non-significant trend in the same direction was observed for the first-person perspective. These findings are largely consistent with previous studies reporting decreased arousal ratings (Poerio et al., 2018; Smejka and Wiggs, 2022; Lohaus et al., 2023) and reduced physiological level of arousal (Tada et al., 2021; Poerio et al., 2018; Engelbregt et al., 2022; Kim et al., 2024; Hozaki et al., 2025) in response to audiovisual ASMR stimulation. Specifically, most of these studies demonstrated a consistent pattern of decreased heart rate, reflecting physiological relaxation and enhanced parasympathetic activity (Poerio et al., 2018; Tada et al., 2021; Engelbregt et al., 2022; Kim et al., 2024; Hozaki et al., 2025). The negligible interaction between visual perspective and audiovisual synchrony suggests that, similar to valence, emotional modulation may not be directly coupled with changes in ASMR intensity.

### Limitations and future directions

The present study has several limitations that warrant further investigation. First, similar to other studies relying on subjective ratings, we quantified participants’ ASMR experiences to infer audiovisual interaction. Future research could incorporate physiological measures to provide objective indices of ASMR intensity. For example, neuroimaging studies have examined physiological responses to ASMR stimuli (Lochte et al., 2018; Smith et al., 2020; Sakurai et al., 2021; Engelbregt et al., 2022; Swart et al., 2022; Assaf et al., 2024). One such study reported significant differences in somatosensory evoked potentials elicited by mild tactile stimulation during the observation of an ASMR video compared with a control video (Assaf et al., 2024). These physiological approaches would help clarify the neural mechanisms underlying ASMR. Second, the effect of visual perspective was coupled with several factors. For instance, actions that are intentionally directed toward the viewer (i.e., viewer-directed attention) were associated with the audiovisual stimuli in the first-person perspective. Moreover, differences in stimulus properties between the two perspectives may also have contributed to the observed effects. Specifically, the absence of an explicit representation of the participant’s own head in the first-person condition may have facilitated embodiment, whereas the presence of a visually distinct dummy head in the third-person condition may have weakened embodiment. The relative contributions of perceived proximity, viewer-directed attention, and embodiment should be examined in future studies, for example by using parametrically controlled or personalized 3D-rendered heads to disentangle visual appearance from visual perspective.

It has been proposed that bodily touch is a strong ASMR trigger and that individuals who experience ASMR also report greater enjoyment and affective responses to social touch, linking ASMR phenomenology to affective tactile sensations (e.g., Gillmeister et al., 2022; Liu and Kondo, 2025). An important future direction would be to investigate multisensory interactions among audition, vision, and touch during social interactions in close proximity. Recent studies have shown that tactile perception of textures resembling human body parts elicits greater pleasantness than other textures in the general population (Pasqualotto et al., 2020; Kitada et al., 2021), possibly contributing to emotional bonding with others (Suvilehto et al., 2019). By contrast, this tactile preference is reduced in individuals with autism spectrum disorder (ASD), that is, in those with higher AASP sensory sensitivity scores than typically developed control (Makita et al., 2025). Interestingly, our findings in exploratory analyses appear to contrast with these touch-related findings, because participants with higher sensory sensitivity in the present study tended to be more responsive to the synchrony × perspective interaction of pleasantness (Table 1). Future studies on tri-sensory interactions involving vision, audition, and touch in close social contexts may help elucidate how individual differences in sensory processing, such as those observed in ASD, influence social interaction and contribute to the avoidance of tactile engagement with others (Fukuoka et al., 2025).

## Conclusion

In conclusion, we examined the influence of audiovisual synchrony and visual perspective on ASMR intensity and its associated emotional responses. We observed a supra-additive interaction between audiovisual synchrony and visual perspective; ASMR intensity was significantly higher in the synchronous than in the asynchronous condition when the stimuli were presented from a first-person perspective. Consistent with the literature on visuo-tactile body illusions, this finding highlights the importance of first-person perspective in modulating the effects of audiovisual synchrony on ASMR.

## Data Availability

The raw data supporting the conclusions of this article will be made available by the authors, without undue reservation.

## Footnotes

1
https://osf.io/g92wa

2
https://www.youtube.com/watch?v=ChKGbkLOokA

3
https://gorilla.sc/

4
https://cran.r-project.org/

5
https://osf.io/q9f2p

## References

[ref1] Ahuja N. K. (2013). It feels good to be measured: clinical role-play, Walker Percy, and the tingles. Perspect. Biol. Med. 56, 442–451. doi: 10.1353/pbm.2013.0022, 24375123

[ref2] Assaf N. Fernandes Soares M. Cardini F. (2024). An investigation of the somatosensory engagement during autonomous sensory meridian response: an ERP study. Biol. Psychol. 193:108961. doi: 10.1016/j.biopsycho.2024.108961, 39644963

[ref3] Atilgan H. Koi J. X. J. Wong E. Laakso I. Matilainen N. Pasqualotto A. . (2023). Functional relevance of the extrastriate body area for visual and haptic object recognition: a preregistered fMRI-guided TMS study. Cereb. Cortex Commun. 4:tgad005. doi: 10.1093/texcom/tgad005, 37188067 PMC10176024

[ref4] Baron-Cohen S. Wheelwright S. Skinner R. Martin J. Clubley E. (2001). The autism-spectrum quotient (AQ): evidence from Asperger syndrome/high-functioning autism, males and females, scientists and mathematicians. J. Autism Dev. Disord. 31, 5–17. doi: 10.1023/A:1005653411471, 11439754

[ref5] Barratt E. L. Davis N. J. (2015). Autonomous sensory meridian response (ASMR): a flow-like mental state. PeerJ 3:e851. doi: 10.7717/peerj.851, 25834771 PMC4380153

[ref6] Barratt E. L. Spence C. Davis N. J. (2017). Sensory determinants of the autonomous sensory meridian response (ASMR): understanding the triggers. PeerJ 5:e3846. doi: 10.7717/peerj.3846, 29018601 PMC5633022

[ref7] Bastiaansen J. A. Thioux M. Keysers C. (2009). Evidence for mirror systems in emotions. Philos. Trans. R. Soc. Lond. Ser. B Biol. Sci. 364, 2391–2404. doi: 10.1098/rstb.2009.0058, 19620110 PMC2865077

[ref8] Blakemore S. J. Bristow D. Bird G. Frith C. Ward J. (2005). Somatosensory activations during the observation of touch and a case of vision-touch synaesthesia. Brain 128, 1571–1583. doi: 10.1093/brain/awh500, 15817510

[ref9] Botvinick M. Cohen J. (1998). Rubber hands “feel” touch that eyes see. Nature 391:756. doi: 10.1038/35784, 9486643

[ref10] Brown C. Dunn W. (2002). Adolescent/Adult Sensory Profile. San Antonio, TX: Psychological Corp.

[ref11] Carey M. Crucianelli L. Preston C. Fotopoulou A. (2019). The effect of visual capture towards subjective embodiment within the full body illusion. Sci. Rep. 9:39168. doi: 10.1038/s41598-019-39168-4, 30814561 PMC6393432

[ref12] Csikszentmihalyi M. (1990). Flow: The Psychology of Optimal Experience. New York: Harper & Row.

[ref13] Engelbregt H. J. Brinkman K. van Geest C. C. E. Irrmischer M. Deijen J. B. (2022). The effects of autonomous sensory meridian response (ASMR) on mood, attention, heart rate, skin conductance and EEG in healthy young adults. Exp. Brain Res. 240, 1727–1742. doi: 10.1007/s00221-022-06377-9, 35511270 PMC9142458

[ref14] Fahey S. Santana C. Kitada R. Zheng Z. (2019). Affective judgement of social touch on a hand associated with hand embodiment. Q J Exp Psychol (Hove) 72, 2408–2422. doi: 10.1177/1747021819842785 30895891

[ref15] Faul F. Erdfelder E. Lang A. G. Buchner A. (2007). G*power 3: a flexible statistical power analysis program for the social, behavioral, and biomedical sciences. Behav. Res. Methods 39, 175–191. doi: 10.3758/BF03193146, 17695343

[ref16] Fredborg B. Clark J. Smith S. D. (2017). An examination of personality traits associated with autonomous sensory meridian response (ASMR). Front. Psychol. 8:247. doi: 10.3389/fpsyg.2017.00247, 28280478 PMC5322228

[ref17] Fredborg B. K. Clark J. M. Smith S. D. (2018). Mindfulness and autonomous sensory meridian response (ASMR). PeerJ 6:e5414. doi: 10.7717/peerj.5414, 30123716 PMC6086079

[ref18] Fredborg B. K. Clark J. M. Smith S. D. (2021). The effects of autonomous sensory meridian response (ASMR) on mood, attention, heart rate, skin conductance and EEG in healthy young adults. Front. Psychol. 12:663072. doi: 10.3389/fpsyg.2021.663072 PMC9142458 35511270

[ref19] Friston K. J. Ashburner J. Kiebel S. J. Nichols T. E. Penny W. D. (2007). Statistical Parametric Mapping: The Analysis of Functional brain Images. London: Academic Press.

[ref20] Fukuoka A. Kitada R. Makita K. Makino T. Sakakihara N. Nummenmaa L. . (2025). Reduced relationship-specific social touching and atypical association with emotional bonding in autistic adults. Mol. Autism. 16:31. doi: 10.1186/s13229-025-00666-0, 40420226 PMC12107774

[ref21] Gillmeister H. Succi A. Romei V. Poerio G. L. (2022). Touching you, touching me: higher incidence of mirror-touch synaesthesia and positive (but not negative) reactions to social touch in autonomous sensory meridian response. Conscious. Cogn. 103:103380. doi: 10.1016/j.concog.2022.103380, 35853396

[ref22] Hales A. H. (2024). One-tailed tests: let's do this (responsibly). Psychol. Methods 29, 1209–1218. doi: 10.1037/met0000610, 37917504

[ref23] Honda S. Ishikawa Y. Konno R. Imai E. Nomiyama N. Sakurada K. . (2020). Proximal binaural sound can induce subjective frisson. Front. Psychol. 11:316. doi: 10.3389/fpsyg.2020.00316, 32194479 PMC7062710

[ref24] Hozaki D. Ezaki T. Poerio G. L. Kondo H. M. (2025). More relaxing than nature? The impact of ASMR content on psychological and physiological measures of parasympathetic activity. Neurosci Conscious 2025:niaf012. doi: 10.1093/nc/niaf012, 40342555 PMC12060867

[ref25] Janik McErlean A. B. Banissy M. J. (2017). Assessing individual variation in personality and empathy traits in self-reported autonomous sensory meridian response. Multisens. Res. 30, 601–613. doi: 10.1163/22134808-00002571 31287086

[ref26] Jones M. R. Daniels A. Igelström K. Suvilehto J. Morrison I. (2025). Tingle-eliciting audiovisual properties of autonomous sensory meridian response (ASMR) videos. Multisens. Res. 38, 427–452. doi: 10.1163/22134808-bja10159 41167257

[ref27] Kim Y. Cho A. Lee H. Whang M. (2024). Impact of sound and image features in ASMR on emotional and physiological responses. Appl. Sci. 14:10223. doi: 10.3390/app142210223

[ref28] Kirk R. E. (2013). Experimental Design: Procedures for the Behavioral Sciences. Thousand Oaks, CA: SAGE Publications.

[ref29] Kitada R. Johnsrude I. S. Kochiyama T. Lederman S. J. (2010). Brain networks involved in haptic and visual identification of facial expressions of emotion: an fMRI study. NeuroImage 49, 1677–1689. doi: 10.1016/j.neuroimage.2009.09.014, 19770059

[ref30] Kitada R. Ng M. Tan Z. Y. Lee X. E. Kochiyama T. (2021). Physical correlates of human-like softness elicit high tactile pleasantness. Sci. Rep. 11:16510. doi: 10.1038/s41598-021-96044-w, 34389767 PMC8363669

[ref31] Kitada R. Okamoto Y. Sasaki A. T. Kochiyama T. Miyahara M. Lederman S. J. . (2013). Early visual experience and the recognition of basic facial expressions: involvement of the middle temporal and inferior frontal gyri during haptic identification by the early blind. Front. Hum. Neurosci. 7:7. doi: 10.3389/fnhum.2013.00007, 23372547 PMC3556569

[ref32] Koumura T. Nakatani M. Liao H. I. Kondo H. M. (2021). Dark, loud, and compact sounds induce frisson. Q J Exp Psychol (Hove) 74, 1140–1152. doi: 10.1177/1747021820977174, 33176602 PMC8107501

[ref33] Kovacevich A. Huron D. (2018). Two studies of autonomous sensory meridian response (ASMR): the relationship between ASMR and music-induced frisson. Empir. Musicol. Rev. 13, 40–63. doi: 10.18061/emr.v13i1-2.6012

[ref34] Liu R. Kondo H. M. (2025). Affective touch sensitivity shapes tingling intensity in autonomous sensory meridian response (ASMR) experiences. Sci. Rep. 15:34974. doi: 10.1038/s41598-025-19082-8, 41057465 PMC12504523

[ref35] Lochte B. C. Guillory S. A. Richard C. A. H. Kelley W. M. (2018). An fMRI investigation of the neural correlates underlying the autonomous sensory meridian response (ASMR). Bioimpacts 8, 295–304. doi: 10.15171/bi.2018.32, 30397584 PMC6209833

[ref36] Lohaus T. Yuksekdag S. Bellingrath S. Thoma P. (2023). The effects of autonomous sensory meridian response (ASMR) videos versus walking tour videos on ASMR experience, positive affect and state relaxation. PLoS One 18:e0277990. doi: 10.1371/journal.pone.0277990, 36598891 PMC9812311

[ref37] Maeno A. Kajimura S. (2022). The role and power of visual trigger on the experience of autonomous sensory meridian response (ASMR). Res Square [Preprint. doi: 10.21203/rs.3.rs-2384295/v1

[ref38] Mahady A. Takac M. De Foe A. (2023). What is autonomous sensory meridian response (ASMR)? A narrative review and comparative analysis of related phenomena. Conscious. Cogn. 109:103477. doi: 10.1016/j.concog.2023.103477, 36806854

[ref39] Makita K. Kitada R. Makino T. Sakakihara N. Fukuoka A. Kosaka H. (2025). Atypical tactile preferences in autism spectrum disorder: reduced pleasantness responses to soft objects resembling human body parts. Psychiatry Clin. Neurosci. 79, 319–326. doi: 10.1111/pcn.13808, 40071820 PMC12131205

[ref40] Maselli A. Slater M. (2013). The building blocks of the full body ownership illusion. Front. Hum. Neurosci. 7:83. doi: 10.3389/fnhum.2013.00083, 23519597 PMC3604638

[ref42] Pasqualotto A. Ng M. Tan Z. Y. Kitada R. Kadosh R. C. (2020). Tactile perception of pleasantness in relation to perceived softness. Sci. Rep. 10:11189. doi: 10.1038/s41598-020-68034-x, 32636415 PMC7341757

[ref43] Petkova V. I. Khoshnevis M. Ehrsson H. H. (2011). The perspective matters! Multisensory integration in ego-centric reference frames determines full-body ownership. Front. Psychol. 2:35. doi: 10.3389/fpsyg.2011.00035, 21687436 PMC3108400

[ref44] Poerio G. L. Blakey E. Hostler T. J. Veltri T. (2018). More than a feeling: autonomous sensory meridian response (ASMR) is characterized by reliable changes in affect and physiology. PLoS One 13:e0196645. doi: 10.1371/journal.pone.0196645, 29924796 PMC6010208

[ref45] Poerio G. Mank S. Hostler T. (2022). The awesome as well as the awful: heightened sensory sensitivity predicts the presence and intensity of autonomous sensory meridian response (ASMR). J. Res. Pers. 97:104183. doi: 10.1016/j.jrp.2021.104183

[ref46] Poerio G. L. Osman F. Todd J. Kaur J. Jones L. Cardini F. (2023). From the outside in: ASMR is characterised by reduced interoceptive accuracy but higher sensation seeking. Multisens. Res. 36, 661–681. doi: 10.1163/22134808-bja10108 37758236

[ref47] Rubin M. (2021). When to adjust alpha during multiple testing: a consideration of disjunction, conjunction, and individual testing. Synthese 199, 10969–11000. doi: 10.1007/s11229-021-03276-4

[ref48] Sakurai N. Ohno K. Kasai S. Nagasaka K. Onishi H. Kodama N. (2021). Induction of relaxation by autonomous sensory meridian response. Front. Behav. Neurosci. 15:761621. doi: 10.3389/fnbeh.2021.761621, 34916914 PMC8669134

[ref49] Shimokura R. (2022). Sound quality factors inducing the autonomous sensory meridian response. Audiol Res 12, 574–584. doi: 10.3390/audiolres12050056, 36285913 PMC9598278

[ref50] Singer T. Seymour B. O’doherty J. Kaube H. Dolan R. J. Frith C. D. (2004). Empathy for pain involves the affective but not sensory components of pain. Science 303, 1157–1162. doi: 10.1126/science.1093535, 14976305

[ref51] Smejka T. Wiggs L. (2022). The effects of autonomous sensory meridian response (ASMR) videos on arousal and mood in adults with and without depression and insomnia. J. Affect. Disord. 301, 60–67. doi: 10.1016/j.jad.2021.12.015, 34915083

[ref52] Smith S. D. Fredborg B. K. Kornelsen J. (2020). Functional connectivity associated with five different categories of autonomous sensory meridian response (ASMR) triggers. Conscious. Cogn. 85:103021. doi: 10.1016/j.concog.2020.103021, 32987225

[ref53] Suvilehto J. T. Nummenmaa L. Harada T. Dunbar R. I. M. Hari R. Turner R. . (2019). Cross-cultural similarity in relationship-specific social touching. Proc. Biol. Sci. 286:20190467. doi: 10.1098/rspb.2019.0467, 31014213 PMC6501924

[ref54] Swart T. R. Banissy M. J. Hein T. P. Bruna R. Pereda E. Bhattacharya J. (2022). ASMR amplifies low frequency and reduces high frequency oscillations. Cortex 149, 85–100. doi: 10.1016/j.cortex.2022.01.004, 35189396

[ref55] Tada K. Ezaki T. Kondo H. M. (2021). The autonomous sensory meridian response activates the parasympathetic nervous system. Res Square [Preprint. doi: 10.21203/rs.3.rs-4543053/v1

[ref56] Tada K. Hasegawa R. Kondo H. M. (2022). Sensitivity to everyday sounds: ASMR, misophonia, and autistic traits. Jpn. J. Psychol. 93, 263–269. doi: 10.4992/jjpsy.93.21319

[ref57] Terashima H. Tada K. Kondo H. M. (2024). Predicting tingling sensations induced by autonomous sensory meridian response (ASMR) videos based on sound texture statistics: a comparison to pleasant feelings. Philos. Trans. R. Soc. Lond. Ser. B Biol. Sci. 379:20230254. doi: 10.1098/rstb.2023.0254, 39005038 PMC11444235

[ref58] Wagenmakers E. J. Wetzels R. Borsboom D. van der Maas H. L. Kievit R. A. (2012). An agenda for purely confirmatory research. Perspect. Psychol. Sci. 7, 632–638. doi: 10.1177/1745691612463078, 26168122

[ref59] Zhang G. L. Li A. S. Miao C. G. He X. Zhang M. Zhang Y. (2018). A consumer-grade LCD monitor for precise visual stimulation. Behav. Res. Methods 50, 1496–1502. doi: 10.3758/s13428-018-1018-7 29532446

