# Supplementary material for: First-person visual perspective enhances audiovisual synchrony effects in autonomous sensory meridian response
Source: Front Psychol. 2026 Mar 27;17:1740614. doi: 10.3389/fpsyg.2026.1740614 (PMC13066313; doi:10.3389/fpsyg.2026.1740614)
Supplement: Supplementary file 1 [file Data_Sheet_1.PDF]

Supplementary Table 1 Descriptive statistics of ASMR ratings for each stimulus (Synchronous conditions)

	1st person perspective					3rd person perspective				
Stimulus type	Glass bottles	News paper	Adhesive tape	Paper cutting	Mean (across stimuli)	Glass bottles	News paper	Adhesive tape	Paper cutting	Mean (across stimuli)
Intensity										
Mean	5.14	6.58	5.10	5.57	5.60	4.48	5.73	4.55	5.06	4.95
SEM	0.30	0.32	0.30	0.31	0.27	0.28	0.34	0.28	0.29	0.25
Valence										
Mean	4.64	5.79	5.77	5.64	5.46	4.30	5.65	5.95	5.77	5.42
SEM	0.31	0.31	0.21	0.23	0.17	0.19	0.26	0.16	0.16	0.11
Arousal										
Mean	4.76	5.24	4.23	4.56	4.70	4.68	5.32	3.79	4.23	4.50
SEM	0.24	0.30	0.23	0.22	0.17	0.18	0.25	0.19	0.22	0.15

Supplementary Table 2 Descriptive statistics of ASMR ratings for each stimulus (Asynchronous conditions)

	1st person perspective					3rd person perspective				
Stimulus type	Glass bottles	News paper	Adhesive tape	Paper cutting	Mean (across stimuli)	Glass bottles	News paper	Adhesive tape	Paper cutting	Mean (across stimuli)
Intensity										
Mean	4.86	6.02	4.63	5.15	5.17	4.44	5.51	4.39	4.79	4.78
SEM	0.27	0.33	0.28	0.29	0.25	0.30	0.35	0.28	0.25	0.25
Valence										
Mean	4.13	5.19	5.42	4.95	4.92	4.00	4.96	5.26	4.99	4.80
SEM	0.24	0.29	0.21	0.22	0.15	0.24	0.30	0.20	0.19	0.16
Arousal										
Mean	5.08	5.36	4.24	4.85	4.88	4.83	5.11	4.39	4.60	4.73
SEM	0.21	0.25	0.18	0.17	0.13	0.16	0.22	0.18	0.18	0.12
